# Metabolism of human pluripotent stem cells and differentiated cells for regenerative therapy: a focus on cardiomyocytes

**DOI:** 10.1186/s41232-021-00156-9

**Published:** 2021-02-01

**Authors:** Sho Tanosaki, Shugo Tohyama, Yoshikazu Kishino, Jun Fujita, Keiichi Fukuda

**Affiliations:** 1 Department of Cardiology, Keio University School of Medicine, Shinjuku, Tokyo, 160-8582 Japan; 2 Department of Emergency and Critical Care Medicine, Keio University School of Medicine, Shinjuku, Tokyo, 160-8582 Japan

**Keywords:** Metabolism, Pluripotent stem cells, Cardiomyocytes, Regenerative therapy

## Abstract

Pluripotent stem cells (PSCs) exhibit promising application in regenerative therapy, drug discovery, and disease modeling. While several protocols for differentiating somatic cells from PSCs exist, their use is limited by contamination of residual undifferentiated PSCs and immaturity of differentiated somatic cells.

The metabolism of PSCs differs greatly from that of somatic cells, and a distinct feature is required to sustain the distinct properties of PSCs. To date, several studies have reported on the importance of metabolism in PSCs and their derivative cells. Here, we detail advancements in the field, with a focus on cardiac regenerative therapy.

## Background

Human PSCs (hPSCs) are capable of self-renewal, proliferation, and differentiation into cells of the three germ layers, and show promising application in drug discovery, disease modeling, and regenerative therapy for diseases resistant to conventional medical therapies. Effective regenerative therapy is contingent on certain considerations, including the establishment of human-induced PSCs (hiPSCs) from somatic cells, expansion of hiPSCs, cell differentiation, removal of residual undifferentiated PSCs, and maturation of differentiated cells. Utilizing the metabolic features of PSCs and their derivative cells is a promising approach to address these considerations, as metabolic intervention is typically a cost-effective and relatively simple solution. To date, numerous studies have demonstrated close relationships between metabolism and cell phenotype, with metabolism and phenotype often affecting one another. This review details the current metabolic understanding of hPSCs and their derivatives, and compares them to mouse PSCs (mPSCs) and cancer cells to clarify similarities and differences among these cells.

## Metabolism of undifferentiated hPSCs

### Glucose

**Fig. 1:**
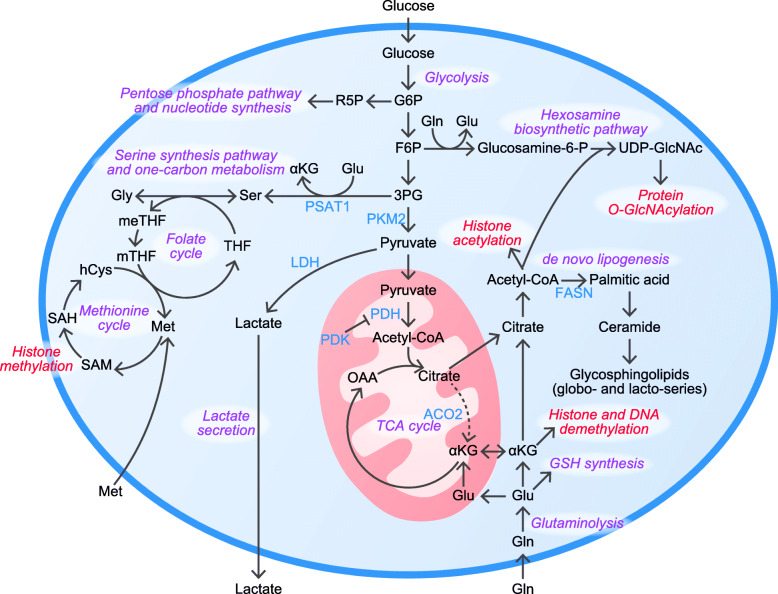
Metabolic features of hPSCs. hPSCs consume glucose for glycolysis, nucleotide synthesis, nonessential amino acid synthesis, and hexosamine biosynthesis. The hexosamine pathway produces UDP-GlcNAc, which serves as a substrate for protein O-GlcNAcylation. The entry of pyruvate into the TCA cycle is inhibited by reduced activity of PDH by PDK. Pyruvate is reduced to lactate to replenish nicotinamide adenine dinucleotide (NAD^+^) to maintain glycolytic activity. Pyruvate, which enters the mitochondrial matrix, is converted to acetyl-CoA and is utilized for citrate production. In hPSCs, the TCA cycle enzyme aconitase 2 is downregulated and conversion into αKG is limited. Citrate transported to the cytosol is converted back to acetyl-CoA, which is used for histone acetylation, de novo fatty acid synthesis, and hexosamine biosynthetic pathway. Fatty acids are utilized for various complex lipids, including glycosphingolipids. To replenish the latter part of the TCA cycle (i.e., αKG to oxaloacetate), hPSCs consume glutamine. Glutamine also plays a key role in glutathione synthesis, the hexosamine biosynthetic pathway, and the serine synthesis pathway. α-KG is required for histone and DNA demethylation. mPSCs consume threonine to produce SAM, but because threonine dehydrogenase is a pseudogene in hPSCs, hPSCs consume methionine to produce SAM. SAM is used for histone methylation. αKG, α-ketoglutarate; ACO2, aconitase 2; F6P, fructose 6-phosphate; FASN, fatty acid synthase; G6P, glucose 6-phosphate; Gln, glutamine; Glu, glutamate; Glucosamine-6-P, glucosamine-6-phosphate; Gly, glycine; GSH, glutathione; hCys, homocysteine; LDH, lactate dehydrogenase; Met, methionine; meTHF, 5,10-methylenetetrahydrofolate; mTHF, 5-methyltetrahydrofolate; PDH, pyruvate dehydrogenase; PDK, pyruvate dehydrogenase kinase; PKM2, pyruvate kinase M2; PSAT1, phosphoserine aminotransferase 1; R5P, ribose 5-phosphate; SAH, *S*-adenosyl homocysteine; SAM, *S*-adenosyl methionine; Ser, serine; TCA cycle, tricarboxylic acid cycle; THF, tetrahydrofolic acid; UDP-GlcNAc, uridine diphosphate N-acetylglucosamine

Rapidly proliferating cells, such as cancer cells, show activated glycolysis even under sufficient oxygen. This phenomenon observed in cancer cells was described by Otto Warburg nearly a century ago, and was thus named the Warburg effect [1]. A possible advantage of the Warburg effect is that it produces sufficient biomass to enable rapid proliferation, that is, production of nucleotides, nonessential amino acids, and lipids. A similar phenomenon is observed in PSCs, which show rapid proliferation similar to cancer cells. This was first described in mPSCs, and later in hPSCs [2, 3] (Fig. 1). Concordant with this report, during reprogramming from somatic cells to hiPSCs, metabolic profiles dramatically change from an oxidative state to a glycolytic state [4]. Studies comparing the gene expression profiles of hPSCs and somatic cells have demonstrated an upregulation of glycolytic genes, including hexokinase 2, with concomitant downregulation of pyruvate dehydrogenase, in hPSCs, which limits entry of glucose-derived pyruvate to the tricarboxylic (TCA) cycle [3]. Interestingly, hexokinase 2 and pyruvate kinase M2 are regulated by Oct4, and their overexpression limits differentiation in mPSCs [5]. Moreover, pyruvate kinase M2 positively upregulates the expression of Oct4 through its regulator activity, suggesting positive feedback in pluripotency and active glycolysis in mPSCs [6].

Reprogramming of mouse embryonic fibroblasts (MEFs) into mouse induced PSCs (miPSCs) results in the transformation of structurally (and functionally) mature mitochondria into immature mitochondria, with a concomitant increase in expression of glycolytic genes, consumption of glucose, and production of lactate. Of note, the rise in glycolytic enzymes precedes that of pluripotent markers [7]. While inhibition of glycolysis by 2-deoxy-d-glucose limits reprogramming efficiency, stimulation of glycolysis by high glucose improves it [7]. In addition, hypoxia-inducible factor 1-alpha (HIF1α) and hypoxia-inducible factor 2-alpha (HIF2α) are upregulated in the early stages of reprogramming in both hiPSCs and miPSCs [8]. HIF1α upregulates pyruvate dehydrogenase kinase, which inhibits pyruvate dehydrogenase activity, preventing the entry of pyruvate into the TCA cycle and activation of glycolysis [9].

As noted above, glycolysis is upregulated by reprogramming into induced PSCs (iPSCs). However, transient upregulation of estrogen-related receptor alpha/gamma (ERRα/γ) and their co-activators, peroxisome proliferator-activated receptor gamma coactivator 1-alpha/beta (PGC-1α/β) with a concomitant transient burst in oxidative phosphorylation (OXPHOS) are observed during reprogramming of hiPSCs and miPSCs [10].

### Hexosamine

The glycolytic intermediate metabolite fructose-6-phosphate is channeled to the hexosamine biosynthetic pathway (HBP) via glutamine-fructose-6-pshophate aminotransferase 1, the first and rate-limiting enzyme of HBP. Uridine diphosphate N-acetylglucosamine (UDP-GlcNAc) is produced by the HBP from glucose, glutamine, acetyl-CoA, and uridine diphosphate, and hence serves as a nutrient sensor for these metabolites by acting as a substrate for O-GlcNAcylation, one of the post-translational protein modifications affecting several catalytic and intracellular signaling pathways. Notably, the core pluripotency marker Oct4 is O-GlcNAcylated [11]. Subsequent studies have demonstrated that disruption of O-GlcNAcylation represses the transcriptional activity of Oct4 in mice [12] and humans [13]. Likewise, elevated O-GlcNAc levels caused by inhibition of O-GlcNAc hydrolase delay mouse embryonic stem cell (mESC) differentiation [14].

### Glutamine and alpha-ketoglutarate

The TCA cycle is a central hub linking the metabolism of glucose, amino acids, and fatty acids. Metabolites derived from these metabolic pathways enter the TCA cycle, and TCA cycle metabolites are utilized for biosynthesis of other metabolites. One example of the utilization of TCA cycle metabolites is citrate, which is used for de novo fatty acid synthesis. In cancer cells, to refuel the TCA cycle, glutamine-derived α-ketoglutarate (αKG) enters the TCA cycle and replenishes oxaloacetate [15]. Furthermore, glutamine-derived malate is metabolized to pyruvate via malic enzyme 1 (NADP-dependent malic enzyme) to meet the high demand for NAPDH, which is also required as a cofactor for de novo fatty acid synthesis [15].

Similar to in cancer cells, glutamine plays key roles in hPSCs, which also exhibit increased de novo fatty acid synthesis. Interestingly, mitochondrial proteins aconitase 2 and isocitrate dehydrogenase 2/3, the enzymes that convert citrate to αKG, are downregulated in hPSCs. The physiological significance of downregulation of these enzymes is uncertain, but it may contribute to the activation of de novo fatty acid synthesis by damming citrate. Since aconitase 2 and isocitrate are downregulated in hPSCs, to sustain the metabolites of the latter part of the TCA cycle, that is, αKG to oxaloacetate, anaplerosis of the TCA cycle by glutamine-derived αKG is essential for the survival of hPSCs [16].

In addition to anaplerosis of the TCA cycle, glutamine is one of the substrates for glutathione (GSH) synthesis, and glutamine supports the stability of OCT4 in hPSCs by sustaining the production of GSH. In the absence of glutamine, endogenous GSH decreases, thereby increasing the oxidation of OCT4 cysteine residues, which results in OCT4 degradation [17].

Moreover, glutamine affects the epigenetic status of mESCs. A study of mESCs revealed that naïve mESCs rapidly consume exogenous glutamine and maintain a high αKG/succinate ratio to promote histone/DNA demethylation. αKG is a cofactor of JmjC domain-containing histone demethylation protein family (JHDM family) and ten-eleven translocation methylcytosine dioxygenase (TET). Both demethylation of trimethylated lysine-27 on histone H3 (H3K27me3) by the JHDM family and demethylation of DNA by TET are regarded as epigenetic modifications of active gene transcription [18].

These studies reveal the importance of glutamine and its metabolites in the survival and maintenance of pluripotency.

### Tryptophan and kynurenine

Tryptophan, one of the essential aromatic amino acids, exhibits versatile roles in PSCs. Its metabolite, kynurenine, acts as a signaling molecule targeting the aryl hydrocarbon receptor (AHR), and two inconsistent studies in mESCs and hPSCs have been reported; mESCs maintain the AHR in a repressive state, and cells that failed to maintain their repressive state showed reduced levels of Oct4 and Sox2 [19]. However, in hPSCs, kynurenine stimulates binding of the AHR to putative promoter and enhancer regions of OCT4 and NANOG [20]. Studies on the roles of tryptophan and kynurenine are scarce and require further investigation.

### S-adenosylmethionine, methionine, and one-carbon metabolism

mESCs catabolize threonine by threonine dehydrogenase and generate glycine and acetyl-CoA, which are utilized in one-carbon metabolism via the glycine cleavage system and TCA cycle, respectively [21]. Furthermore, these metabolites are utilized in the production of *S*-adenosylmethionine (SAM), which serves as a methyl donor in various biological reactions. Depletion of threonine from culture medium decreases SAM levels and leads to decreased trimethylation of histone H3 lysine 4 (H3K4me3), a methylation marker for the active state [22], resulting in increased differentiation.

A similar phenomenon can be observed in hPSCs. However, since threonine dehydrogenase is a pseudogene in humans, hPSCs cannot utilize threonine for the production of SAM; instead, hPSCs utilize methionine to maintain a high level of SAM. Transient deprivation of methionine decreases H3K4me3 and promotes cell differentiation, and long-term deprivation leads to p53-mediated cell death [23].

However, a study on naïve human embryonic stem cells (hESCs) showed that SAM needs to be controlled at low levels. In naïve hESCs, nicotinamide *N*-methyltransferase is upregulated, along with its product 1-methylnicotinamide. This enables a reduction of SAM levels and maintains a low level of H3K27me3, an epigenetic marker for repressive gene transcription and required for a naïve state [24]. Therefore, the role of SAM and its influence on epigenetic status in PSCs may be context dependent and must be interpreted with caution.

### Lipids

De novo fatty acid synthesis is a process in which saturated fatty acid is synthesized from acetyl-CoA. Fatty acids are further elongated by elongase and/or desaturated by desaturases, and are utilized for the generation of glycerolipids (i.e., phosphatidylcholine, phosphatidylethanolamine, phosphatidylserine, and triacylglycerol) and sphingolipids (i.e., sphingomyelin and ceramide), which serve as the building blocks of phospholipid bilayers or energy storage. De novo fatty acid synthesis is upregulated in certain types of tumor cells to enable rapid cellular proliferation [25]. As for PSCs, during reprogramming of MEFs into miPSCs, levels of acetyl-CoA carboxylase and fatty acid synthase (FASN), the enzymes involved in de novo fatty acid synthesis, are upregulated. Pharmacological inhibition of these two enzymes results in decreased reprogramming efficiency [26]. Lipid synthesis, including de novo fatty acid synthesis and cholesterol synthesis, is regulated by sterol regulatory element-binding transcription factor 1 (Srebf1). Therefore, given that iPSCs activate lipid synthesis, one might expect that Srebf1 has a positive role in the reprogramming of somatic cells to iPSCs. Overexpression of Srebf1 reportedly enhanced reprogramming efficiency into miPSCs, while inhibition of Srebf1 reduced this efficiency. However, this regulatory effect does not arise from its regulation of lipid metabolism, but arises from its interaction with c-Myc, resulting in enhanced expression of pluripotent genes [27]. Interestingly, de novo fatty acid synthesis sustains mitochondrial dynamics in two ways in miPSCs, by producing fatty acids required for phospholipids, which are important components of the mitochondrial membrane, and by consuming fatty acid synthesis substrate acetyl-CoA. Consumption of acetyl-CoA prevents acetylation of mitochondrial fission 1 protein (FIS1) and acetylation-mediated ubiquitin-proteasome degradation of FIS1, leading to enhanced mitochondrial fission [28].

De novo fatty acid synthesis is not only crucial for reprogramming into miPSCs, but is also crucial for the survival of hPSCs. Knockdown of FASN, the final enzyme involved in de novo fatty acid synthesis, induces apoptosis in undifferentiated hPSCs. Inhibitors of FASN are similarly able to induce apoptosis in these cells. Interestingly, hiPSC-derived somatic cells, including cardiomyocytes (CMs), neurons, and hepatocytes, were not susceptible to FASN inhibition. These data suggest that FASN inhibition may be suitable for clinical regenerative medicine [29]. In undifferentiated hPSCs, oleate synthesis via SCD1 is upregulated; thus, its inhibition enables selective elimination of hPSCs [30].

Although mESCs and miPSCs share similar metabolic features, there are some differences. There is no notable difference in the metabolism of carbohydrates, hydroxy acid, free fatty acids, and the pentose phosphate pathway between mESCs and miPSCs. However, there are significant differences in phosphatidylcholine and phosphatidylethanolamine lipid structures, essential and non-essential amino acids, and polyamines [31].

Interestingly, hPSCs are able to modulate their glycolytic dependency under different culture conditions. hPSCs show a reduced dependency on glycolysis when cultured in the presence of feeder cells, and a greater dependency when cultured in feeder-free conditions [32]. This metabolic difference arises from the lipids contained in the culture media, since the lipids produced by feeder cells are consumed by the hPSCs and lipid supplementation is able to recapitulate the metabolic features of hPSCs grown with feeder cells in hPSCs without feeder cells. This data supports the idea that activated glycolysis provides biomass for cell proliferation. Another study reported a decreased dependency on glycolysis in primed hESCs cultured with MEFs [33]. Although this study did not specify the metabolite(s) needed to recapitulate the phenomenon, lipids are one of the candidate metabolites involved in this phenomenon. In addition, a recent report suggests that the lipid composition of the medium can alter the naïveness of hPSCs. Lipid deprivation in culture media skews hPSCs into a naïve-to-primed intermediate state [34]. Lipid deprivation-induced inhibition of extracellular signal-regulated kinase (ERK) signaling and skewed hPSCs toward the naïve-to-primed intermediate state.

Sphingolipids are a class of lipids containing sphingosine as a basal structure. De novo synthesis of sphingolipids starts from serine palmitoyl transferase, which consumes serine and palmitoyl-CoA as substrates. Ceramide is then glycosylated and diverges into various glycosphingolipids. The composition of glycosphingolipids varies between cell types, and hPSCs show a unique composition, with the glycosphingolipids stage-specific embryonic antigen (SSEA)-4 and SSEA-3 serving as PSC markers. Liang et al. reported the unique composition of glycosphingolipids, and that this composition changes during differentiation [35]. In hESCs, globo- and lacto-series glycosphingolipids are abundant. Along with embryoid body differentiation, these two types of glycosphingolipids decrease and are replaced with ganglio-series glycosphingolipids.

### Mitochondria

Several studies have demonstrated that the mitochondria of PSCs are small and functionally immature compared to somatic cells [2, 36]. However, a study by Zhang et al. reported that hPSCs contain functional respiratory complexes and expression of uncoupling protein 2 (UCP2) in hPSCs decouple activated glycolysis and mitochondrial glucose oxidation [36]. Similarly, another group has reported that UCP2 exports TCA cycle metabolites, such as malate and oxaloacetate, from mitochondria [37].

Mitochondrial dynamics are crucial for the establishment of pluripotency and various mechanisms involved in the reprogramming of MEFs. In MEFs, OSKM (*Oct4*, *Sox2*, *Klf4*, and *Myc*) reprogramming causes Myc-induced cell proliferation, which leads to a decrease in mitochondria per cell, while OSK (*Oct4*, *Sox2*, and *Klf4*) reprogramming decreases mitochondria through another mechanism. In OSK reprogramming, mitochondrial mass initially increases and subsequently decreases through BNIP3L/NIX-mediated mitophagy [38].

Above all, it should be noted that it is the balance of mitochondrial dynamics (i.e., mitochondrial fission and fusion) that is important for pluripotency, and excess mitochondrial fission limits pluripotency. By balancing mitochondrial dynamics, Ca^2+^ homeostasis is maintained; therefore, CaMKII activity is balanced, leading to the accumulation of beta-catenin, which ultimately results in pluripotency in miPSCs [39].

## Metabolism and differentiation

It is apparent that metabolism is not only passively altered during differentiation but also affects the cell fate of hPSCs. Here, we highlight the importance of metabolism in cell fate decision.

### Glucose

**Fig. 2:**
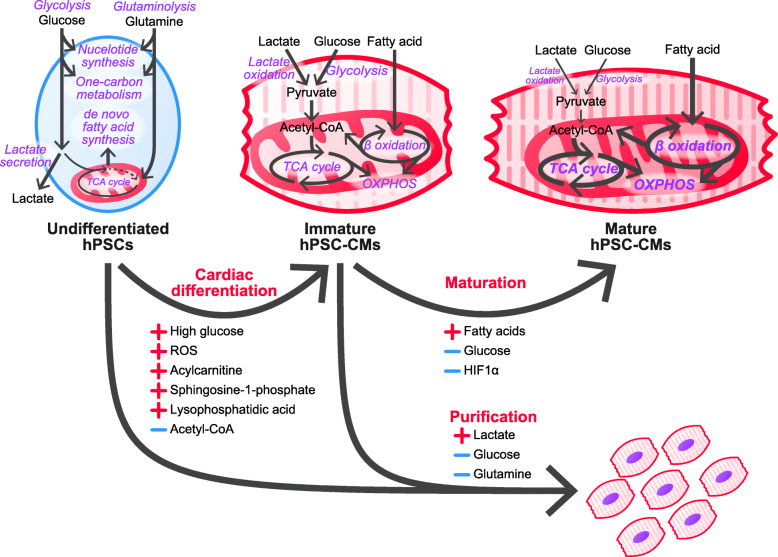
Metabolic regulation of hPSC cardiac differentiation and maturation. Undifferentiated hPSCs upregulate glycolysis, glutaminolysis, nucleotide synthesis, one-carbon metabolism, de novo fatty acid synthesis. Cardiac differentiation is enhanced by high glucose, ROS, fatty acylcarnitine, sphingosine-1-phosphate, and lysophosphatidic acid. On the other hand, glucose-derived acetyl-CoA inhibits differentiation. hPSC-CMs are metabolically immature and rely on lactate oxidation and glycolysis. By growing hPSC-CMs with fatty acids in the absence of glucose, hPSC-CMs metabolically mature and activate β oxidation and OXPHOS. Activation of HIF1α inhibits maturation. hPSC-CMs can be purified by utilizing the metabolic features of undifferentiated hPSCs and hPSC-CMs. Glucose- and glutamine-deprived, lactate-supplemented medium can purify hPSCs and eliminate undifferentiated hPSCs and non-cardiomyocytes. OXPHOS, oxidative phosphorylation

In mESCs, culture conditions containing high glucose enhance cardiac differentiation through reactive oxygen species (ROS) production [40] (Fig. 2). Moreover, the addition of H_2_O_2_ is able to promote the differentiation of mESCs to CMs. Treatment with radical scavengers, such as trolox, pyrrolidine dithiocarbamate, and N-acetylcysteine, showed the opposite effect [41].

While the metabolic switch of PSCs from a glycolytic to an oxidative state during differentiation is well established, the specific timing of the transition is dependent on which lineage the cells are differentiating into. During differentiation to endoderm and mesoderm lineages, their metabolism switches to OXPHOS, but in the early phase of differentiation to ectoderm, high glycolytic flux is maintained, which requires MYC/MYCN in hPSCs [42].

Glycolysis-derived acetyl-CoA affects pluripotency and differentiation in mPSCs. Acetyl-CoA produced by glycolysis is used for histone acetylation, and inhibition of glycolysis decreases histone acetylation levels and promotes differentiation. Supplementation with acetate, which can be converted to acetyl-CoA, could delay differentiation [43].

### Glutamine and αKG

While αKG maintains pluripotency through modification of histone and DNA demethylation, its ability to modify epigenetic status also affects differentiation. αKG promotes early differentiation in hPSCs, and reduced αKG delays differentiation. The accumulation of succinate, which is produced from αKG by histone or DNA demethylases, similarly, delays differentiation. Overall, the αKG/succinate ratio modifies histone methylation status [44].

However, the opposing effect of αKG on differentiation has been reported in a mESC study. There are three major pathways that generate αKG from glutamate, namely, glutamate dehydrogenase, alanine transaminase, and aspartate transaminase. However, phosphoserine aminotransferase 1 (Psat1), a serine synthesis pathway enzyme, also consumes glutamate and generates αKG. In mESCs, αKG produced by Psat1 alters H3K9me3 and H3K36me3 levels, and Psat1-knockdown accelerates differentiation [45].

These studies on the relationship between αKG and differentiation are controversial, but modulation of the serine synthesis pathway may alter one-carbon metabolism and, ultimately, histone and DNA methylation status through the production of SAM. Together with the difference in one-carbon metabolism, especially that of threonine, in mice and humans as described above, the somewhat opposing role of αKG on mouse and human PSCs needs further investigation.

### Lipids

The lipid profile of mESCs shows abundant polyunsaturated fatty acids. Inhibition of polyunsaturated fatty acid metabolism, such as inhibition of Δ6/Δ-desaturase, phospholipase A2, cyclooxygenase 1/2, or lipoxygenase, results in delayed neuronal differentiation, which is defined by persistent expression of *Oct4* and *Nanog*. Moreover, supplementation of the mitochondrial β-oxidation substrate acylcarnitine during differentiation promoted neuronal and cardiac differentiation [46]. This is consistent with reports that ROS promote cardiac differentiation, as mitochondrial β-oxidation produces ROS. In addition, sphingosine-1-phosphate and lysophosphatidic acid, both bioactive lipid species, are able to enhance cardiac differentiation in hiPSCs through nuclear accumulation of β-catenin, the canonical Wnt pathway mediator [47].

As for sphingolipids, during differentiation to the ectodermal lineage, globo- and lacto-series glycosphingolipids are replaced with ganglioside-series glycosphingolipids. During differentiation to the endodermal lineage, Gb4 ceramide dominates [48]. It has been reported that the abundance of globo-series glycosphingolipids accounts for the expression of neuronal transcription factors. Globo-series sphingolipids reside in the promoter region of autism susceptibility candidate 2 (AUTS2), and along with neuronal differentiation, reduction in globo-series glycosphingolipids leads to the induction of AUTS2, and AUTS2 activates the production of ganglio-series glycosphingolipids and other neuronal genes [49].

## Exploiting the metabolic signature of hPSCs in regenerative medicine

Regenerative medicine using hPSCs is an attractive therapy for diseases resistant to conventional medical therapies. However, several limitations must be overcome to achieve full clinical application. Among them, tumor formation by residual undifferentiated cells is of great importance, and while several strategies have been proposed to overcome this limitation, many are not ideal for clinical application due to high cost and poor scalability. Our research group has developed a strategy based on the distinct metabolic features of hPSCs; the different metabolic features of undifferentiated hPSCs and hPSC-derived CMs (hPSC-CMs), and their potential application, will here-after be discussed (Fig. 2).

### hPSC-CMs

hPSC-CMs are immature and resemble that of the fetus in terms of their size, sarcomere structure, contractile activity, electrophysiological activity, mitochondrial structure, and metabolism [50]. Mature CMs rely mainly on fatty acid oxidation for energy production, while fetal CMs rely mainly on glycolysis and lactate oxidation [51]. Although the metabolism of hPSC-CMs is similar to that of fetal CMs and immature compared to adult CMs, differentiation of hPSCs to hPSC-CMs is accompanied by a transition from aerobic glycolysis to mitochondrial oxidation, and it greatly differs from that of hPSCs and hPSC-derived non-CMs, which are generated during cardiac differentiation [52]. Therefore, the difference in metabolism can be exploited to purify hPSC-CMs after differentiation from hPSCs. Undifferentiated hPSCs and hPSC-derived non-CMs rely largely on glucose and glutamine metabolism, and their deprivation leads to cell death. hPSC-CMs are able to survive under glucose and glutamine deprivation conditions, in the presence of lactate, whereby hPSC-CMs consume lactate to fuel the TCA cycle and produce energy. Therefore, in glucose- and glutamine-deprived, lactate-supplemented culture medium, undifferentiated hPSCs and hPSC-derived non-CMs cannot survive, whereas hPSC-CMs can survive, thus allowing for their purification. This method is particularly promising in cardiac regeneration therapy using hPSC-CMs because it has advantages in cost, ease-of-use, and scalability [16, 53, 54].

As described previously, the mitochondria of undifferentiated hPSCs are small and immature. Mitochondrial metabolism (i.e., the TCA cycle, fatty acid oxidation, and OXPHOS) in hPSC-CMs is immature compared to adult CMs, but hPSC-CMs contain larger amounts of mitochondria than undifferentiated hPSCs. Moreover, mitochondria of hPSC-CMs are larger in size and more mature than those of undifferentiated hPSCs. Therefore, fluorescence-assisted cell sorting of hPSC-CMs by tetramethylrhodamine methyl ester perchlorate (TMRM) staining enables efficient purification of hPSC-CMs [55].

The immaturity of hPSC-CMs may limit their application in regenerative therapy, drug discovery, and disease modeling. Long-term culture of hPSC-CMs improves maturation, but it has been reported that hPSC-CMs after long-term culture cannot reach the level of maturation of adult CMs [56, 57]. Therefore, to facilitate maturation, several methods have been reported, including culture substrates, mechanical stress, electrical stimulation, and thyroid hormones [58–61]. Of these, metabolism is one of the more promising strategies. Metabolism is an important factor influencing cell properties and can be further exploited in the maturation of hPSC-CMs. Fatty acid oxidation is a hallmark metabolic feature of adult CMs, and glucose-deprived and fatty acids (palmitic acid and oleic acid)-supplemented culture medium can facilitate maturation [62, 63]. However, excess saturated fatty acids (i.e., palmitic acid) may cause lipotoxicity [64]. Galactose supplementation to glucose-deprived and fatty acid-supplemented medium ameliorated lipotoxicity caused by saturated fatty acids and enhanced maturation [65]. Cultures with glucose-containing medium upregulate the HIF1α-lactate dehydrogenase A axis and leads to active glycolysis. By inhibiting HIF1α or lactate dehydrogenase A with small molecules, structural, metabolic, and electrophysiological maturation was achieved [65]. Another study reported that high-glucose culture conditions inhibit the maturation of hPSC-CMs via the pentose phosphate pathway [66]. Overall, these findings underscore the importance of metabolism and the need for optimization of culture conditions to facilitate the maturation of hPSC-CMs.

## Conclusions

In this study, we have detailed several studies reporting on the distinct metabolic features of hPSCs and how metabolism can affect the properties of hPSCs and their derivatives. While the clinical application of regenerative medicine using hPSC-derived somatic cells is gaining momentum, various cost and safety concerns remain to be addressed. Exploiting the metabolic characteristics governing cells at different stages may serve as an effective strategy to mitigate these issues.

## Data Availability

Not applicable.

## Abbreviations

αKG: α-ketoglutarate
AHR: Aryl hydrocarbon receptor
AUTS2: Autism susceptibility candidate 2
ERRα/γ: Estrogen-related receptor alpha/gamma
ERK: Extracellular signal-regulated kinase
ESC: Embryonic stem cell
FASN: Fatty acid synthase
GSH: Glutathione
HBP: Hexosamine biosynthetic pathway
hESC: Human embryonic stem cell
HIF: Hypoxia-inducible factor
hiPSC: Human induced pluripotent stem cells
hPSC: Human pluripotent stem cell
iPSC: Induced pluripotent stem cell
JHDM family: JmjC domain-containing histone demethylation protein family
mESC: Mouse embryonic stem cell
miPSC: Mouse induced pluripotent stem cell
mPSC: Mouse pluripotent stem cell
NAD^+^: Nicotinamide adenine dinucleotide
OXPHOS: Oxidative phosphorylation
PGC-1α/β: Peroxisome proliferator-activated receptor gamma coactivator 1-alpha/beta
PSC: Pluripotent stem cell
Srebf1: Sterol regulatory element binding transcription factor 1
SSEA: Stage specific embryonic antigen
TCA cycle: Tricarboxylic acid cycle
TET: Ten-eleven translocation methylcytosine dioxygenase
UDP-GlcNAc: Uridine diphosphate N-acetylglucosamine
